# ﻿One new species and three new records of the leafhopper genus *Agnesiella* Dworakowska (Hemiptera, Cicadellidae, Typhlocybinae) from China

**DOI:** 10.3897/zookeys.1238.153092

**Published:** 2025-05-15

**Authors:** Junjie Wang, Wenya Ma, Yalin Zhang, Min Huang

**Affiliations:** 1 Key Laboratory of Plant Protection Resources and Pest Management of Ministry of Education, College of Plant Protection, Northwest A&F University, Yangling, Shaanxi 712100, China Northwest A&F University Yangling China

**Keywords:** Auchenorrhyncha, identification key, morphology, new record, Typhlocybini

## Abstract

One new species, Agnesiella (Draberiella) geminicruciata**sp. nov.**, is described and illustrated. Agnesiella (D.) lidia, A. (D.) olena and A. (D.) magda are documented from China for the first time. A key to males of all known species of *Agnesiella* from China is provided.

## ﻿Introduction

The leafhopper genus *Agnesiella* Dworakowska (tribe Typhlocybini) is a species-rich group predominantly found in the Oriental region, exhibiting exceptional diversity in southwestern China. *Agnesiella* species are primarily associated with *Alnus* (Betulaceae) as host plants, although records indicate some species utilize *Juglans* (Juglandaceae) (Wang et al. 2024). *Agnesiella* was established by Dworakowska in 1970, with *Typhlocybaaino* Matsumura, 1932 designated as the type species. In the same publication, Dworakowska (1970) transferred three additional species from *Typhlocyba* (*T.giranna*, *T.lyraeformis*, and *T.nitobella*, all Matsumura, 1932) to *Agnesiella*. Subsequently, Dworakowska (1971) erected the subgenus Agnesiella (Draberiella), with *Chikkaballapuraquinquemaculata* Distant, 1918 as its type species, differentiating it from the nominate subgenus by the presence of clustered setae on the pygofer side, typically divided into two groups, a feature absent in the nominate subgenus.

Currently, *Agnesiella* includes 56 described species distributed across the Palaearctic and Oriental regions. The nominate subgenus comprises 13 species, while the subgenus Draberiella contains 43 species (Wang et al. 2024).

In this study, we describe one new species and report three new records of *Agnesiella* from China, bringing the total recognized species count to 57, with 48 species now recorded from China. A taxonomic key to the *Agnesiella* species currently known from China is provided.

## ﻿Material and methods

All specimens examined are deposited in the Entomological Museum, Northwest A&F University, Yangling, China (**NWAFU**). The abdomens and genitalia were treated with hot 10% NaOH solution for 2 minutes to dissolve muscle tissue and stored in glycerine. Morphological observations and illustrations utilized an Olympus SZX10 microscope and an Olympus BH-2 drawing apparatus. Specimens were photographed using a Leica M205 microscope equipped with a Leica DFC425 camera, utilizing the Leica Application Suite (LAS) v. 3.7 software. Final image processing was performed using Adobe Photoshop 2024 (Adobe Systems).

Morphological terminology follows Zhang (1990), except for wing venation, which follows Dworakowska (1993).

## ﻿Taxonomy

### ﻿ Agnesiella

Taxon classificationAnimaliaHemipteraCicadellidae

Dworakowska, 1970

5865643A-FFFB-515A-A593-2B1F578B6BBD

Agnesiella Dworakowska, 1970: 211.

#### Type species.

*Typhlocybaaino* Matsumura, 1932.

#### Diagnosis.

Body with ground color ivory to brownish. Vertex-face junction usually with 2 sesame-like whitish patches; vertex usually with a pair of roundish black patches near the inner margins of the eyes. Pronotum usually with an oval dark longitudinal patch near center of anterior margin, and 1 or 2 pairs of dark lateral patches. Forewing basal 2/3 generally with brownish patches, patches at distal end of ScP+RA and MP’’+CuA’ veins, and 3^rd^ apical cell usually brownish; brochosome area semi-transparent to reddish-brown with patches at both ends brownish to black.

Crown obtusely protruding medially, narrower than pronotum with length about 1/2 that of pronotum, and with anterior and posterior margins approximately parallel. Pronotum with anterior margin arched and posterior margin straight. Apical half of forewing narrowing gradually, with RP and MP’ veins confluent at the base and 3^rd^ apical cell subtriangular. Hind wing transparent with R and MP veins confluent terminally.

Abdominal 2S apodemes developed and usually extending to 5^th^ or 6^th^ abdominal sternite. Male pygofer generally with 2 setal groups; posterior margin generally with digitiform process extending either dorsally or posteriorly, and a small protrusion bearing small rigid setae on upper part. Length of genital valve approximately 1/5 to 1/4 length of subgenital plate. Subgenital plate club-shaped, usually with slightly expanded distal end, outer margin usually narrowed subapically with a protrusion generally bearing few peg-like setae; apical half usually with small rigid setae and long, fine setae. Paramere usually with subapical processes varying in length, number and angle; caudal half with row of microsetae on outer side and row of sensilla pores on inner side. Connective Y-shaped with stem well developed. Aedeagal shaft slender, usually with lamellar or digitiform ventral processes and tooth-like end curling inwards; gonopore apical.

#### Remarks.

This genus shares similarities with *Linnavuoriana* Dlabola, 1958 in the presence of paired spots on the head and pronotum, and the absence of macrosetae on the subgenital plate. However, it is distinguished by a pair of black submarginal spots on the vertex, a process on the posterior margin of the male pygofer, and a process on the ventral margin of the aedeagal shaft.

#### Distribution.

Oriental and Palaearctic regions.

##### ﻿Key to males of *Agnesiella* from China

**Table:** 

1	Setae not clustered on pygofer side (*Agnesiella*)	**2**
–	Setae clustered and usually divided into 2 groups on pygofer side (*Draberiella*)	**13**
2	Pygofer side with a bifurcate process	***A.marginata* Dworakowska**
–	Pygofer side with an unbranched process	**3**
3	Pygofer process without setae	***A.polita* Huang & Zhang**
–	Pygofer process with setae	**4**
4	Paramere without subapical protrusion	***A.recurva* Huang & Zhang**
–	Paramere with subapical protrusion	**5**
5	Paramere with 1 subapical protrusion	**6**
–	Paramere with 2 subapical protrusions	**7**
6	Paramere with a subapical process directed away from apex	***A.hamaculeata* Wang & Huang**
–	Paramere with a subapical process forming an obtuse angle with apex	***A.nigroflava* Wang & Huang**
7	Paramere with apex angle with upper subapical protrusion nearly equal to 90°	***A.juglandis* Chou & Ma**
–	Paramere with apex angle with upper subapical protrusion significantly greater than 90°	**8**
8	Aedeagal shaft with incision on dorsal margin subapically	***A.buysi* Dworakowska**
–	Aedeagal shaft without incision on dorsal margin subapically	**9**
9	Pronotum with dark brown patches on central area near posterior margin	**10**
–	Pronotum without dark brown patches on central area near posterior margin	**12**
10	Pygofer with a long digitiform pygofer process	***A.nitobella* (Matsumura, 1932)**
–	Pygofer with a short horn-like pygofer process	**11**
11	Forewing with patches separately at basal and apical cell areas	***A.lyraeformis* (Matsumura, 1932)**
–	Forewing with patches separately at basal, middle, and cross-veins areas	***A.matsumurai* Dworakowska**
12	Paramere with 2 interlaced subapical protrusions	***A.protensa* Huang & Zhang**
–	Paramere with 2 non-interlaced subapical protrusions	***A.giranna* (Matsumura, 1932)**
13	Pygofer process arising from posteroventral angle	**14**
–	Pygofer process arising from posterior margin	**32**
14	Pygofer process with 2 branches	***A.rita* Dworakowska**
–	Pygofer process unbranched	**15**
15	Paramere with 2 subapical protrusions	***A.roxana* Dworakowska**
–	Paramere with 1 subapical protrusion	**16**
16	Aedeagal shaft without distinct ventral process	**17**
–	Aedeagal shaft with distinct ventral process	**18**
17	Forewing with V-shaped patches in the middle	***A.innota* Yan & Yang**
–	Forewing with separate patches at the basal and cross-vein areas	***A.glabra* Huang & Zhang**
18	Ventral process of aedeagal shaft with 2 branches	**19**
–	Ventral process of aedeagal shaft with 1 branch	**23**
19	Paramere with a subapical protrusion nearly as long as caudal apex	**20**
–	Paramere with a subapical protrusion distinctly shorter than caudal apex	**21**
20	Two branches of ventral process of aedeagal shaft with a long stalk	***A.furca* Yan & Yang**
–	Two branches of ventral process of aedeagal shaft without a long stalk	***A.mitrata* Huang & Zhang**
21	Two branches of ventral process of aedeagal shaft arising directly from shaft in lateral view	***A.hastata* Wang & Huang**
–	Two branches of ventral process of aedeagal shaft arising separately from plate-like extension in lateral view	**22**
22	Pygofer process closely appressed to posterior margin of pygofer	***A.azra* Dworakowska**
–	Pygofer process not closely appressed to posterior margin of pygofer	***A.bupa* Dworakowska**
23	Ventral process of aedeagal shaft arising from base of the shaft	***A.elongata* Wang & Huang**
–	Ventral process of aedeagal shaft arising from apical half or middle of the shaft	**24**
24	Aedeagal shaft with a distinct plate-like expansion dorsally on subapical part	**25**
–	Aedeagal shaft without a distinct plate-like expansion dorsally on subapical part	**28**
25	Aedeagal shaft with end of ventral process curved ventrally	**26**
–	Aedeagal shaft with end of ventral process curved dorsally	**27**
26	Aedeagal shaft with a short digitiform end of ventral process	***A.erosa* Yan & Yang**
–	Aedeagal shaft with a long digitiform end of ventral process	***A.gaura* Huang & Zhang**
27	Ventral process of aedeagal shaft with a digitiform end	***A.stipitata* Huang & Zhang**
–	Ventral process of aedeagal shaft with a short horn-like end	***A.lata* Huang & Zhang**
28	Ventral process of aedeagal shaft with serrated posterior margin	***A.eleganta* Huang & Zhang**
–	Ventral process of aedeagal shaft with smooth posterior margin	**29**
29	Aedeagal shaft with a short, horn-like ventral process at the middle	***A.magda* Dworakowska**
–	Aedeagal shaft with a long digitiform ventral process directed ventrally	**30**
30	Pygofer side without long fine setae near posterior margin	***A.latusa* Yan & Yang**
–	Pygofer side with long fine setae near posterior margin	**31**
31	Aedeagal shaft with subapical ventral margin irregularly undulated	***A.sinuata* Yan & Yang**
–	Aedeagal shaft with subapical ventral margin not undulated	***A.apiculata* Huang & Zhang**
32	Pygofer process multi-branched	**33**
–	Pygofer process unbranched	**36**
33	Pygofer process with 3 branche	***A.tridigitata* Huang & Zhang**
–	Pygofer process with 2 branches	**34**
34	Ventral process of aedeagal shaft with 2 branches	***A.chelata* Wang & Huang**
–	Ventral process of aedeagal shaft plate-like	**35**
35	Ventral process of aedeagal shaft with smooth posterior margin	***A.lidia* Dworakowska**
–	Ventral process of aedeagal shaft with undulated posterior margin	***A.kamala* Dworakowska**
36	Ventral process of aedeagal shaft multi-branched	**37**
–	Ventral process of aedeagal shaft unbranched	**47**
37	Ventral process of aedeagal shaft with 3 branches	**38**
–	Ventral process of aedeagal shaft with 2 branches	**40**
38	Three branches of ventral process of aedeagal shaft, each arising separately from common stem	***A.longisagittata* Huang & Zhang**
–	Two outer branches of ventral process of aedeagal shaft with common stem separate from innermost branch	**39**
39	Aedeagal shaft with innermost branch of ventral process extending to apex of the shaft	***A.savita* Dworakowska**
–	Aedeagal shaft with innermost branch of ventral process extending to subapical part of the shaft	***A.irma* Dworakowska**
40	All branches of ventral process of aedeagal shaft directed dorsally	**41**
–	Two branches of ventral process of aedeagal shaft separately directed dorsally and ventrally	**43**
41	Aedeagal shaft with apex of innermost branch of ventral process extending beyond apex of the shaft	***A.olena* Dworakowska**
–	Aedeagal shaft with innermost branch of ventral process extending to subapical part of the shaft	**42**
42	Ventral process of aedeagal shaft with two adjacent branches	***A.biprotrusa* Huang & Zhang**
–	Ventral process of aedeagal shaft with two relatively separated branches	***A.exigua* Huang & Zhang**
43	Aedeagal shaft with serrated middle part of dorsal margin	***A.ela* Dworakowska**
–	Aedeagal shaft with smooth dorsal margin	**44**
44	Pygofer with a short, horn-like pygofer process	**45**
–	Pygofer with a long digitiform pygofer process	**46**
45	Two branches of ventral process of aedeagal shaft, each arising separately from common stem	***A.xantha* Yan & Yang**
–	Two branches of ventral process of aedeagal shaft, each arising separately from shaft (Figs 25, 26)	***A.geminicruciata* sp. nov.**
46	Ventral process of aedeagal shaft with a lower branch curved toward the side of shaft	***A.digita* Yan & Yang**
–	Ventral process of aedeagal shaft without a lower branch curved toward the side of shaft	***A.quinquemaculata* (Distant, 1918)**
47	Ventral process of aedeagal shaft extending to subapical part of shaft	***A.singuliprotrusa* Huang & Zhang**
–	Ventral process of aedeagal shaft extending to apex of shaft	***A.galeata* Wang & Huang**

### ﻿ Agnesiella (Draberiella) lidia

Taxon classificationAnimaliaHemipteraCicadellidae

Dworakowska

47D4AB01-A487-5F52-B193-A41C1290AFB9

Figs 1–4 , 17

Agnesiella (D.) lidia Dworakowska, 1977: 38.

#### Specimens examined.

China • 4 ♂♂, 2 ♀♀; Guangxi Prov., Langping, Mt. Cengwanglao; 106°22'50.73"E, 24°28'53.56"N; 1430 m; 23 Jul. 2021; X. Zhou coll.; NWAFU • 3 ♂♂; Sichuan Prov., Shimian, Liziping National Nature Reserve; 102°23'5.11"E, 29°1'20.76"N; 2000 m; 9 Aug. 2021; J.J. Wang coll.; NWAFU • 5 ♂♂, 3 ♀♀; Yunnan Prov., Baoshan, Gaoligong National Nature Reserve; 98°48'0.02"E, 25°18'5.93"N; 2000 m; 16 Jun. 2022; J.J. Wang coll.; NWAFU • 4 ♂♂, 2 ♀♀; Yunnan Prov., Dali, Mt. Cangshan; 100°8'5.46"E, 25°41'54.57"N; 2200 m; 24 Jun. 2022; J.J. Wang coll.; NWAFU.

**Figures 1–16.**
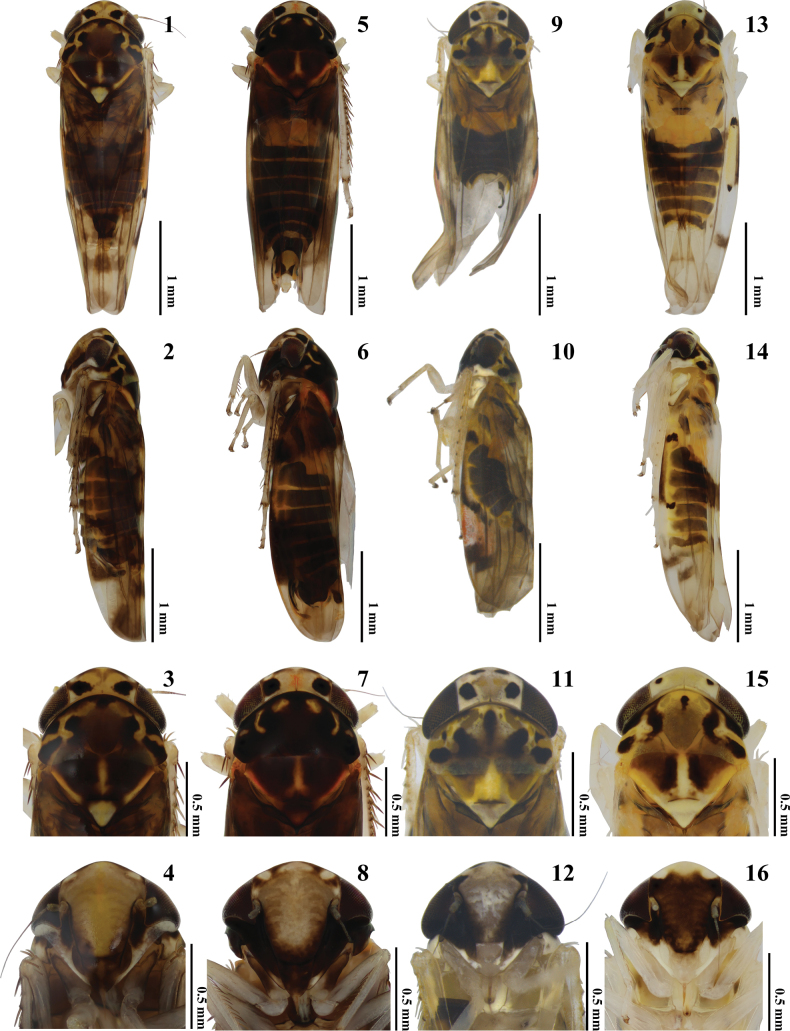
External morphology of *Agnesiella* species **1, 5, 9, 13** habitus, dorsal view **2, 6, 10, 14** habitus, lateral view **3, 7, 11, 15** head and thorax, dorsal view **4, 8, 12, 16** face **1–4***A.lidia* rec. nov. **5–8***A.olena* rec. nov. **9–12***A.magda* rec. nov. **13–16***A.geminicruciata* sp. nov. Scale bars: 1.0 mm (**1, 2, 5, 6, 9, 10, 13, 14**); Scale bars: 0.5 mm (**3, 4, 7, 8, 11, 12, 15, 16**).

#### Distribution.

China (new record) (Guangxi, Sichuan, Yunnan) , Vietnam.

### ﻿ Agnesiella (Draberiella) olena

Taxon classificationAnimaliaHemipteraCicadellidae

Dworakowska

EEC0AAEA-CB7A-5485-BCE3-AECCB177BB16

Figs 5–8 , 18

Agnesiella (D.) olena Dworakowska, 1977: 37.

#### Specimens examined.

China • 3 ♂♂; Xizang Autonomous Region, Rikaze, Jilong Port; 85°22'47.29"E, 28°16'38.35"N; 1920 m; 20 Jul. 2022; Q.Q. Xue coll.; NWAFU • 5 ♂♂, 2 ♀♀; Yunnan Prov., Baoshan, Gaoligong National Nature Reserve; 98°46'57.29"E, 24°49'10.46"N; 2050 m; 18 Jun. 2022; J.J. Wang coll.; NWAFU • 3 ♂♂, 1 ♀; Yunnan Prov., Dali, Mt. Cangshan; 100°8'5.46"E, 25°41'54.57"N; 2200 m; 25 Jun. 2022; J.J. Wang coll.; NWAFU • 5 ♂♂, 3 ♀♀; Yunnan Prov., Lijiang, Xinzhu; 99°27'21.40"E, 27°17'41.71"N; 2500 m; 3 Jul. 2022; J.J. Wang coll.; NWAFU.

#### Distribution.

China (new record) (Xizang, Yunnan), Vietnam.

### ﻿ Agnesiella (Draberiella) magda

Taxon classificationAnimaliaHemipteraCicadellidae

Dworakowska

63B4F853-ADFE-54F1-88B3-3E46F1BD03B5

Figs 9–12 , 19

Agnesiella (D.) magda Dworakowska, 1982: 132.

#### Specimens examined.

**China • 2** ♂♂; **Xizang Autonomous Region, Rikaze**, Jilong; 85°19'40.26"E, 28°23'37.74"N; 2700 m; 17 Jul. 2022; Q.Q. Xue coll.; NWAFU.

#### Distribution.

China (new record) (Xizang), India.

### ﻿ Agnesiella (Draberiella) geminicruciata

Taxon classificationAnimaliaHemipteraCicadellidae

Wang & Huang sp. nov.

661326B5-3538-5EB4-A6B9-6F71C8B9A62A

https://zoobank.org/CE204A13-B17D-4EC7-9A2C-D3559DC50685

Figs 13–16 , 20 , 21–26

#### Description.

Body largely yellowish (Figs 13, 14). Face mostly dark brown, frontoclypeal area with dark brown transverse stripes, lorum ivory (Fig. 16). Vertex yellowish-brown medially, remaining parts yellowish, with a pair of blackish-brown patches. Pronotum with some blackish-brown patches, median area light brown; triangles dark brown, scutellum yellowish (Fig. 15). Forewing with one brown patch each at middle and apical part in basal 2/3, apical 1/3 smoky brown, brochosome area pale yellow with a blackish-brown patch at each end (Fig. 20).

**Figures 17–20.**
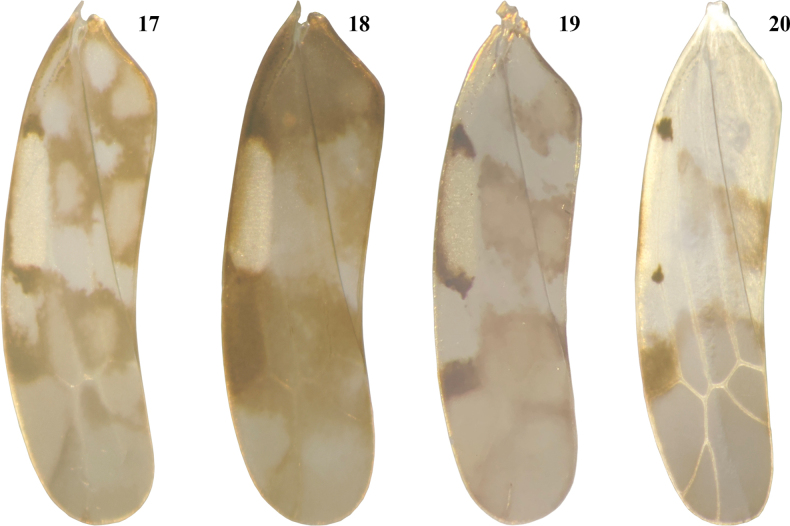
Forewing of *Agnesiella* species **17***A.lidia* rec. nov. **18***A.olena* rec. nov. **19***A.magda* rec. nov. **20***A.geminicruciata* sp. nov.

**Figures 21–26.**
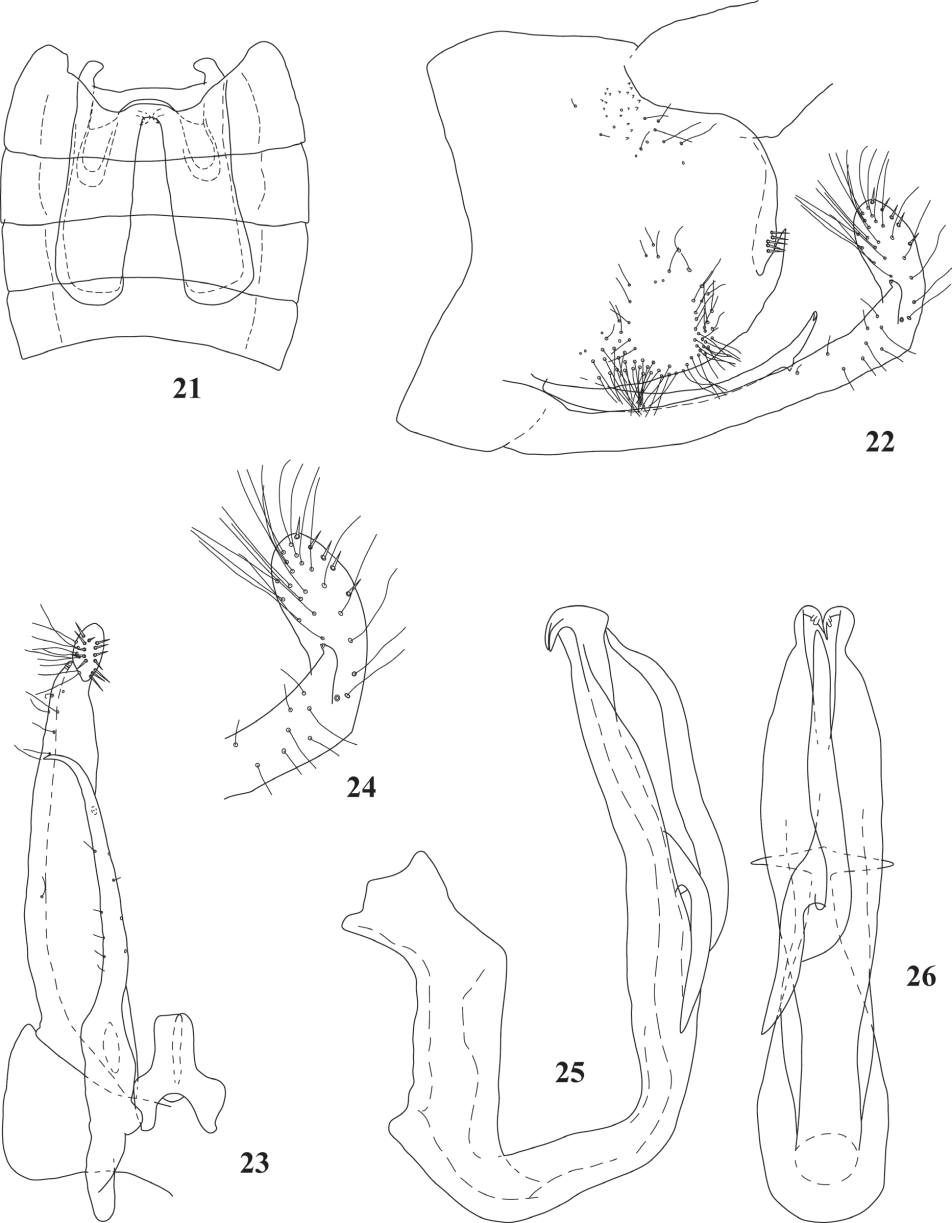
A. (D.) geminicruciata Wang & Huang, sp. nov. **21** abdominal apodemes **22** genitalia capsule, lateral view **23** paramere, connective and subgenital plate, dorsal view **24** apical 1/3 of subgenital plate, lateral view **25** aedeagus, lateral view **26** aedeagus, posterior view.

Abdominal apodemes extending nearly to middle of 6^th^ abdominal sternite (Fig. 21).

***Male genitalia*.** Male pygofer with two longitudinal bands of long fine setae medially near ventral margin, and posterior band more elongate; posterior margin with a short horn-like pygofer process on the lower part, a medial cluster of small rigid setae (Fig. 22). Subgenital plate with expanded distal part, bearing some long fine setae and small rigid setae; a distinct protrusion subapically with peg-like setae apically (Figs 22–24). Paramere slender, with apex curving distinctly outward and a small subapical tooth on ventral margin (Figs 22, 23). Connective with central lobe (Fig. 23). Aedeagal shaft relatively straight, with lamellate lateral expansions (Figs 25, 26); ventral margin with two intersecting processes at the middle: right process longer, extending to shaft apex, and left process curves leftward to the shaft in posterior view (Fig. 26).

#### Specimens examined.

***Holotype***: China • ♂; Yunnan Prov., Puer, Ailaoshan National Nature Reserve; 101°15'55.50"E, 24°16'32.83"N; 2200 m; 6 Jun. 2022; J.J. Wang; NWAFU. ***Paratypes***: 2 ♂♂, 1 ♀, same data as for holotype • 5 ♂♂, 3 ♀♀; Yunnan Prov., Luchun, Huanglianshan National Nature Reserve; 102°32'33.71"E, 23°2'2.24"N; 2050 m; 10 Jun. 2023; L. Lu; NWAFU.

#### Measurement.

Males, 3.15–3.28 mm (including wing).

#### Etymology.

This specific epithet is derived from the Latin words “*gemini*” and “*crux*”, referring to the two intersecting processes on the ventral margin of the aedeagal shaft (Figs 25, 26).

#### Remarks.

This new species closely resembles *A.xantha* Yan & Yang in the male genitalia, but can be distinguished by two intersecting ventral processes on the aedeagal shaft, each originating independently from the shaft (Figs 25, 26).

## Supplementary Material

XML Treatment for Agnesiella

XML Treatment for Agnesiella (Draberiella) lidia

XML Treatment for Agnesiella (Draberiella) olena

XML Treatment for Agnesiella (Draberiella) magda

XML Treatment for Agnesiella (Draberiella) geminicruciata

## ﻿Additional information

### Author ORCIDs

Junjie Wang https://orcid.org/0000-0002-6611-1075

Wenya Ma https://orcid.org/0009-0004-6405-4435

Yalin Zhang https://orcid.org/0000-0002-1204-9181

Min Huang https://orcid.org/0000-0001-7621-4863

### Data availability

All of the data that support the findings of this study are available in the main text.
